# Phase diagrams for biophysical fitness landscape design

**DOI:** 10.1101/2025.09.10.675274

**Published:** 2026-03-20

**Authors:** Vaibhav Mohanty, Eugene I. Shakhnovich

**Affiliations:** 1 Department of Chemistry and Chemical Biology, Harvard University, Cambridge, MA 02138; 2 Harvard/MIT MD-PhD Program, Harvard Medical School, Boston, MA 02115 and Massachusetts Institute of Technology, Cambridge, MA 02139; 3 Program in Health Sciences and Technology, Harvard Medical School, Boston, MA 02115 and Massachusetts Institute of Technology, Cambridge, MA 02139

## Abstract

Populations evolve toward the top of fitness landscapes in the way that physical particles experience forces toward the bottom of a potential energy landscape. We recently introduced fitness landscape design (FLD) as a means of customizing the shape of a protein’s biophysical fitness landscape by using antibody sequences and concentrations as tunable control parameters. Until now, previous evidence for the feasibility of FLD has been numerical and simulated. Here, we derive an explicit analytical theory for the FLD phase diagram, providing tight bounds on the designability frontiers describing the extent to which fitnesses of different protein sequences can be tuned independently of each other. We then leverage a recently published experimental dataset of binding affinities between over 62,000 antibody variants and each of three glycoprotein antigens to construct experimental FLD phase diagrams, which show close agreement with theory. These results support the feasibility of engineering quantitatively programmable fitness landscapes for laboratory protein evolution experiments.

## Introduction.

Evolution of a population is often viewed as a semi-random “survival of the fittest” process in which natural selection contributes a driving force towards increased fitness over time, while genotype mutations facilitate exploration of the sequence space [1–4]. Mathematically, absolute fitness F(s) is defined as the growth rate of a strain or species s; i.e., dn(s)/dt=F(s)n(s), where n(s) is the number of individuals of type s [1]. First introduced by Sewall Wright more than 90 years ago [5], the mapping from genotype sequence s to fitness F(s) is visually represented as a mountain-like structure termed a *fitness landscape*, which has served as a useful tool for understanding evolutionary trajectories. The structure of a fitness landscape can be affected by intrinsic factors, such as protein mutations which impact organism survivability, or interactions with the environment, such as protein-protein interactions between a cell surface protein and extracellular peptides [6–13].

**FIG. 1.**
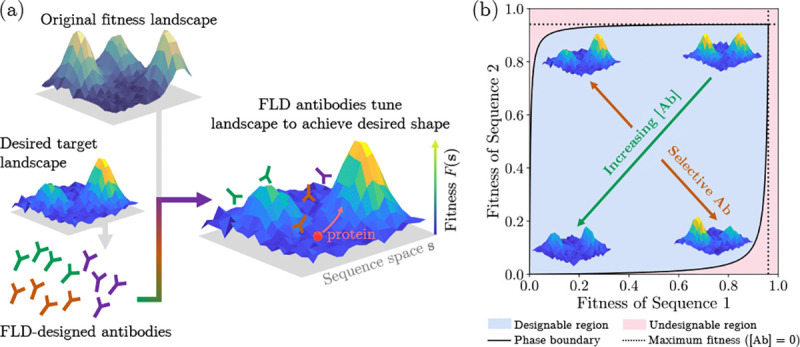
(a) Schematic for fitness landscape design, which uses antibodies used as tunable control parameters to customize the biophysical fitness landscape of a target protein by suppressing different sequence fitnesses with user-defined penalties. (b) Schematic FLD phase diagram, showing how the fitnesses of two different sequences (tops of the two peaks) can be suppressed relative to one another.

We recently introduced *fitness landscape design* (FLD) [14], which is the task of molding a target protein’s biophysical fitness landscape into a desired structure by adjusting the biophysical interactions between the target protein and a set of antibodies which bind to the target protein. Essentially, in FLD, antibodies are tunable control parameters which reshape a protein’s biophysical fitness landscape according to a user-specified template landscape (Figure 1(a)). Some physically inspired studies have studied control of evolutionary dynamics purely from a dynamical systems perspective or by regulating drug dosage or chemical environments in real time [15–18], but FLD [14] is the first to suggest that the fitness landscape underlying evolution can itself be shaped into a quantitatively predictable desired structure. As an application, we showed that our FLD algorithms can discover cross-reactive antibodies which proactively suppress the fitness trajectories of viral escape mutants [14].

FLD relies on the idea that antibody-protein interactions have sufficient flexibility such that different target protein sequences can experience a wide range of possible fitnesses. For example, flooding a system with antibodies—even weakly binding ones—will generally lead to low fitnesses for all strains. Unsurprisingly, having zero antibodies in solution with the viral bulk culture will lead to the maximum possible fitneses for each strain. However, numerical evidence has shown that it is possible to discover selective antibodies which can suppress some fitnesses but not others, allowing for many degrees of freedom in tuning fitness landscape shapes by finding appropriate antibody sequences [14]. These possibilities are described by the schematic FLD phase diagram in Figure 1(b), which characterizes what fitness landscape assignments are “designable” or “undesignable.”

In this work, we derive an explicit analytical theory for the shape of the FLD phase diagram, in agreement with previous numerical estimates [14]. We then leverage a recently published experimental dataset of over 62, 000 antibodies’ binding affinities to each of three different influenza surface antigens to construct the first experimental FLD phase diagrams, demonstrating excellent agreement with theory and supporting the notion that FLD is experimentally realizable.

### Analytical theory.

We now develop an analytical theory for the boundary between the *designable* and *undesignable* regions of the FLD phase diagram. For viral surface protein evolution, biophysical properties—particularly, binding affinities—of viral-host cell binding and viral-antibody are quantitatively predictive of the viral strains’ fitnesses. A statistical mechanics-based biophysical model of viral fitness which we showed can be analytically derived from chemical reaction kinetics between viral, host cell, and antibody proteins [14] aptly captures the empirical fitness data. The viral fitness can be analytically expressed as

(1) $$F(s)\approx \frac{k_{\text{rep}}\left(N_{o}\;-\;1\right)N_{\text{ent}}[\text{H}]e^{-\beta \Delta G_{\text{H}}(s)}}{C_{0}\;+\;[\text{H}]e^{-\beta \Delta G_{\text{H}}(s)}\;+\;\sum_{n}\left[{\text{Ab}}_{n}\right]e^{-\beta \Delta G_{\text{Ab}}^{\left(n\right)}(s)}},$$

where $k_{\text{rep}}$ is a virion’s microscopic rate constant for cell entry and replication, $N_{o}$ is the average number of offspring produced a single replication event, $N_{\text{ent}}$is the number of viral surface proteins which can potentially be used for cell entry, [H] is the total concentration of host cell receptors, $\left[{\text{Ab}}_{n}\right]$is the total concentration of the n-th type of antibody, $\Delta G_{\text{H}}(s)$is the binding affinity between the viral entry protein and host cell receptor, $\Delta G_{\text{Ab}}^{\left(n\right)}$is the binding affinity between the entry protein of viral strain s and the n-th antibody, and β is the inverse temperature. This model has been extensively validated by simulations of viral serial dilution experiments [14, 19], by *in vitro* growth experiments in norovirus [20], and by epidemiological infectivity data for SARS-CoV-2 [21].

To make analytical calculations tractable, we work in the case where we have two target protein sequences (“sequence 1” and “sequence 2”, denoted as $s_{1}$ and $s_{2}$) and only one antibody whose concentration [Ab] and sequence serve as control parameters. We use rescaled fitnesses with constants $k_{\text{rep}}\left(N_{o}-1\right)N_{\text{ent}}=1$ for mathematical convenience. The biophysical fitness model simplifies to

(2) $$F(s)\approx \frac{[\text{H}]e^{-\beta \Delta G_{\text{H}}(s)}}{C_{0}\;+\;[\text{H}]e^{-\beta \Delta G_{\text{H}}(s)}\;+\;[\text{Ab}]e^{-\beta \Delta G_{\text{Ab}}(s)}},$$

for each of the two sequences.

To find the boundaries of the designable region, we ask what are the maximum and minimum fitnesses $F_{2}\equiv F\left(s_{2}\right)$ that sequence 2 could exhibit given a fixed fitness $F_{1}\equiv F\left(s_{1}\right)$ for sequence 1? To calculate the bounds on $F_{2}$ given $F_{1}$, we first rearrange eq. (2)

(3) $$[\text{Ab}]=\frac{[\text{H}]e^{-\beta \Delta G_{\text{H}}(s)}}{e^{-\beta \Delta G_{\text{Ab}}(s)}}\left(\frac{1}{F(s)}-\frac{C_{0}}{[\text{H}]e^{-\beta \Delta G_{\text{H}}(s)}}-1\right).$$

The above equation holds separately for sequences 1 and 2, which would both approximately experience the same antibody concentration in the limit where the antibody concentration great exceeds viral strain concentrations, which is a clinically relevant limit in viral infections such as SARS-CoV-2 in vaccinated individuals [14]. Equating the antibody concentrations gives us

(4) $$\begin{array}{l} \frac{[\text{H}]e^{-\beta \Delta G_{\text{H}}\left(s_{1}\right)}}{e^{-\beta \Delta G_{\text{Ab}}\left(s_{1}\right)}}\left(\frac{1}{F_{1}}-\frac{C_{0}}{[\text{H}]e^{-\beta \Delta G_{\text{H}}\left(s_{1}\right)}}-1\right)\\ =\frac{[\text{H}]e^{-\beta \Delta G_{\text{H}}\left(s_{2}\right)}}{e^{-\beta \Delta G_{\text{Ab}}\left(s_{2}\right)}}\left(\frac{1}{F_{2}}-\frac{C_{0}}{[\text{H}]e^{-\beta \Delta G_{\text{H}}\left(s_{2}\right)}}-1\right), \end{array}$$

and solving for $F_{2}$ in terms of $F_{1}$, we have

(5) $$F_{2}\left(F_{1}\right)={\left[\frac{1}{F_{2}^{\text{free}}}+\rho e^{\beta \Delta }\left(\frac{1}{F_{1}}-\frac{1}{F_{1}^{\text{free}}}\right)\right]}^{-1},$$

where we have defined $F_{i}^{\text{free}}\equiv \frac{[\text{H}]e^{-\beta \Delta G_{\text{H}}\left(s_{i}\right)}}{C_{0}+[\text{H}]e^{-\beta \Delta G_{\text{H}}\left(s_{i}\right)}}$ as the fitness for the “free” protein when antibody concentration is zero, $\Delta \equiv \Delta G_{\text{Ab}}\left(s_{1}\right)-\Delta G_{\text{Ab}}\left(s_{2}\right)$ as the free energy difference between the two sequences’ bound states to the antibody, and $\rho \equiv e^{-\beta \left(\Delta G_{\text{H}}\left(s_{1}\right)-\Delta G_{\text{H}}\left(s_{2}\right)\right)}$, which is equivalent to the ratio of the two sequences’ dissociation constants with the host protein.

Now, we note that host-viral binding affinities $\Delta G_{\text{H}}\left(s_{1}\right)$ and $\Delta G_{\text{H}}\left(s_{2}\right)$ are fixed (because we cannot manually mutate the host receptor protein), but we do have control over the choice of antibody, which is chosen from a library of M antibodies. We work within the approximation that the two antibody-antigen binding free energies $\Delta G_{\text{Ab}}\left(s_{1}\right)$ and $\Delta G_{\text{Ab}}\left(s_{2}\right)$ are chosen from a bi-variate Gaussian distribution:

(6) $$\left(\begin{array}{c} \Delta G_{\text{Ab}}\left(s_{1}\right)\\ \Delta G_{\text{Ab}}\left(s_{2}\right) \end{array}\right)\;\sim \;𝒩\left(\left(\begin{array}{c} \mu_{1}\\ \mu_{2} \end{array}\right),\left(\begin{array}{cc} \sigma_{1}^{2} & r_{12}\sigma_{1}\sigma_{2}\\ r_{12}\sigma_{1}\sigma_{2} & \sigma_{2}^{2} \end{array}\right)\right),$$

where means $\mu_{1}$ and $\mu_{2}$, standard deviations $\sigma_{1}$ and $\sigma_{2}$, and the Pearson correlation $r_{12}\in [-1,1]$ are taken to be be parameters fixed by the choice of the two target antigens $s_{1}$ and $s_{2}$. The antibody we choose is taken from a finite library of size M; for instance, if we consider all combinatorial amino acid variation at L paratope sites, $M=20^{L}$. Like in Derrida’s random energy model (REM) [22, 23], having a finite (even if a large) number of possible samples means that there will be a finite ground state energy. The frontiers of the designable phase will be determined by such extremal antibody samples from the library which maximize the magnitude $|\Delta |=|\Delta G_{\text{Ab}}\left(s_{1}\right)-\Delta G_{\text{Ab}}\left(s_{2}\right)$, the difference between the two antibody-antigen binding free energies. Such antibodies are selective in that they tightly bind to one sequence but not the other, causing one sequence to have low fitness while the other is high.

From the Gaussian approximation, we know that Δ is also Gaussian distributed: $\Delta \;\sim \;𝒩\left(\mu_{\Delta },\sigma_{\Delta }\right)$, where we define $\mu_{\Delta }\equiv \mu_{1}-\mu_{2}$ and $\sigma_{\Delta }=\sqrt{\sigma_{1}^{2}+\sigma_{2}^{2}-2\sigma_{1}\sigma_{2}r_{12}}$. A standard result in classical extreme-value statistics [24] provides an approximation to the mean of the maximum (or minimum) value of Δ in the limit of large M samples:

(7) $$\Delta_{\pm }\approx \mu_{\Delta }\pm \sigma_{\Delta }y_{M},$$

where

(8) $$y_{M}=\sqrt{2\log M}-\frac{\log(4\pi \log M)\;-\;2\gamma }{2\sqrt{2\log M}}+O\left(\frac{1}{\log M}\right),$$

and γ is the Euler-Mascheroni constant. The leading order contribution to $y_{M}$ is exactly the ground state energy of the REM [23], and the sub-leading order term which scales as $O(\log\log M/\sqrt{\log M})$ is related to finite-size corrections to the ground state energy of the REM [25]. Heuristically, we can understand that the Gaussian approximation employed here works because the antibody-antigen complex in the bound state can be treated as a heteropolymer in a folded state, with the variable monomers (or spins, in the REM analogy) consisting of L amino acids on the antibody surface. The equivalence of the spin-glass model of folded heteropolymers to the REM is well-known result in protein folding physics [26]. The antibody for which $\text{Δ}={\text{Δ}}_{-}$ is the “ground state” antibody which selectivelybindsantigensequence1morestronglythanitbinds to sequence 2, compared to any other antibody in the library. Conversely, the $\text{Δ}={\text{Δ}}_{+}$ solution yields an antibody which selectively binds antigen sequence 2 more strongly than it binds to sequence 1, compared to any other antibody in the library.

We can now calculate sequence 2’s maximum possible fitness $F_{2}^{+}\left(F_{1}\right)$ and minimum possible fitness $F_{2}^{-}\left(F_{1}\right)$ given the fitness of sequence 1, which will be obtained from the $\text{Δ}={\text{Δ}}_{-}$ and $\text{Δ}={\text{Δ}}_{+}$ antibodies, respectively

(9) $$F_{2}^{\pm }\left(F_{1}\right)={\left[\frac{1}{F_{2}^{\text{free}}}+\rho e^{\beta \Delta_{\mp }}\left(\frac{1}{F_{1}}-\frac{1}{F_{1}^{\text{free}}}\right)\right]}^{-1}.$$

These designability boundaries—a central result of this work—are valid on the sequence 1 fitness domain $F_{1}\in \left(0,F_{1}^{\text{free}}\right]$ , which corresponds to the sequence 2 fitness range $F_{2}\in \left(0,F_{2}^{\text{free}}\right]$. The exact same boundaries could be obtained by repeating the derivation but instead maximizing or minimizing $F_{1}$ for fixed $F_{2}$, yielding relabeled but equivalent curves $F_{1}^{+}\left(F_{2}\right)=F_{2}^{-}\left(F_{1}\right)$ and $F_{1}^{-}\left(F_{2}\right)=F_{2}^{+}\left(F_{1}\right)$.

**FIG. 2.**
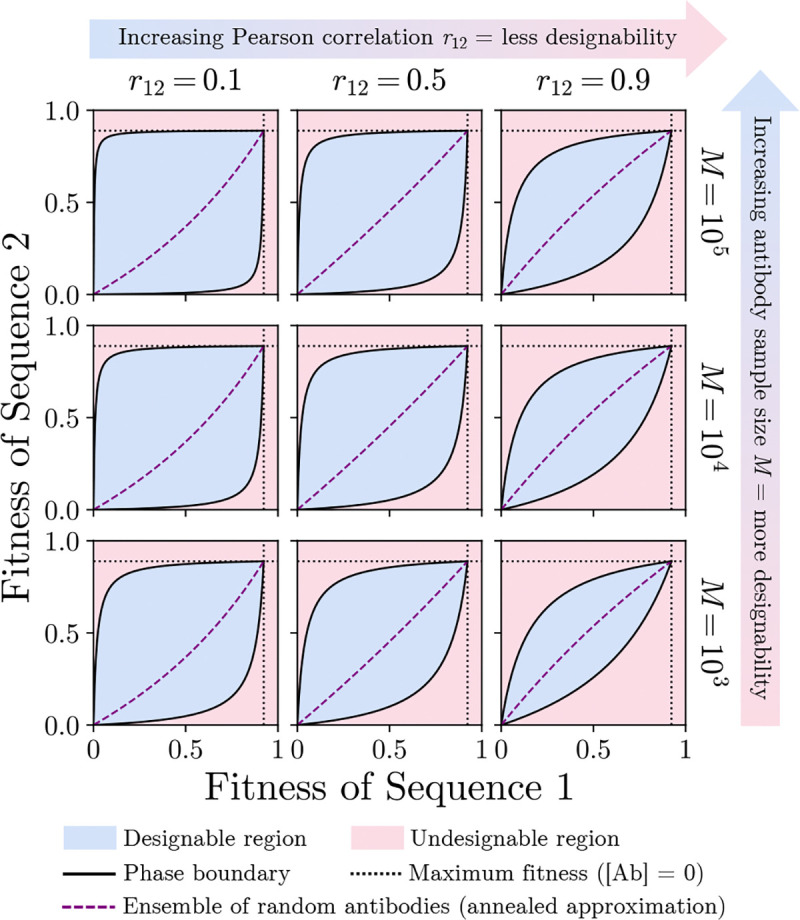
Example theoretical FLD phase diagrams from eq. (9). The area of the designable region expands with decreasing Pearson correlation $r_{12}$ and with increasing antibody library size M.

We plot theoretical phase diagrams using eq. (9) in Figure 2 across a range of antibody library sizes M and Pearson correlations $r_{12}$. The designable region (blue) is generally an asymmetric leaf-shaped region within the unit square whose sharp corners are placed at (0, 0) and $\left(F_{1}^{\text{free}},F_{2}^{\text{free}}\right)$. The two curves $F_{2}^{+}\left(F_{1}\right)$ and $F_{2}^{-}\left(F_{1}\right)$ (black solid lines) respectively outline the top and bottom borders of the designable region, and they converge at two points, representing limiting cases of the antibody concentration: in the limit of no antibodies ([Ab]=0), both fitnesses adopt their “free” limits $F_{1}=F_{1}^{\text{free}}$ and $F_{2}=F_{2}^{\text{free}}$ (black dotted lines). Fitnesses above these “free” limits are unobtainable since antibodies do not enhance antigen-host binding. In the limit of high antibody concentration ([Ab]→∞), we see from the original fitness equation eq. (2) that both fitnesses $F_{1}$, $F_{2}\to 0$. Outside of the designable region is the undesignable region (red), where fitness assignments are unreachable by any choice of antibody. Although we work in the single antibody limit, we can also obtain the estimated fitness curve (purple dashed line) for a large random ensemble of different antibodies with uniform concentrations by taking the annealed approximation $e^{\beta \text{Δ}}\mapsto E\left[e^{\beta \text{Δ}}\right]=e^{\beta \mu_{\text{Δ}}+\frac{1}{2}\beta \sigma_{\text{Δ}}^{2}}$ within eq. (5).

We see in Figure 2 that the designable region expands with increasing antibody library size M and with decreasing Pearson correlation between $\Delta G_{\text{Ab}}\left(s_{1}\right)$ and $\Delta G_{\text{Ab}}\left(s_{2}\right)$. Increasing M also increases the expected maximum/minimum of the free energy samples from the Gaussian distribution in eq. (6). The latter trend makes sense when we contextualize the biophysical meaning of $r_{12}$. Two sequences are likely to have high $r_{12}$ when they are more chemically similar. For example, swapping a small nonpolar amino acid for another on the antigen is difficult for the antibody to chemically distinguish. The binding affinities are likely to be similar, so both sequences’ fitnesses are likely to be similar, which decreases the area of the designable region. Conversely, swapping multiple nonpolar amino acids for charged amino acids can make $r_{12}$ go to zero or even become negative. We now develop an analytical theory quantitatively explain this behavior of the designable region.

The area of the designable region relative to the (trivial) area of the unit square is called the *codesignability score* $\mathrm{CDS}\left(s_{1},s_{2}\right)$ for the two sequences, as defined and computed numerically in ref. [14]. High codesignability score indicates increased flexibility for fitness assignment: an antibody can be found such that sequence 1 fitness can be high while sequence 2 fitness is low—or vice versa—and it is possible to modulate antibody concentration so that the fitnesses are both high or both low. We previously showed that codesignability scores for all pairs of sequences in a larger set are useful for understanding what groups of sequences may have their fitnesses designed together, though all of our computations of codesignability scores were numerical [14]. Here, we can compute codesignability score analytically.

By definition, codesignability score is the area of the designable region, which is computed by finding the area between the $F_{2}^{+}\left(F_{1}\right)$ and $F_{2}^{-}\left(F_{1}\right)$ curves:

(10) $$\mathrm{CDS}\left(s_{1},s_{2}\right)=\int_{0}^{F_{1}^{\text{free}}}\text{d}F_{1}\left[F_{2}^{+}\left(F_{1}\right)-F_{2}^{-}\left(F_{1}\right)\right],$$

which has an exact integral solution:

(11) $$\mathrm{CDS}\left(s_{1},s_{2}\right)=I\left(\rho e^{\beta \Delta_{-}}\right)-I\left(\rho e^{\beta \Delta_{+}}\right),$$

where

(12) $$I(x)\equiv \frac{F_{1}^{\text{free}}F_{2}^{\text{free}}}{{\left(1\;-\;\frac{xF_{2}^{\text{free}}}{F_{1}^{\text{free}}}\right)}^{2}}\left[1-\frac{xF_{2}^{\text{free}}}{F_{1}^{\text{free}}}\left(1-\log\frac{xF_{2}^{\text{free}}}{F_{1}^{\text{free}}}\right)\right].$$

We prove analytically in the End Matter that $\partial \text{CDS}/\partial r_{12}<0$ for any valid $r_{12}$, so codesignability monotonically decreases with Pearson correlation $r_{12}$, suggesting that chemically similar protein antigens are harder to assign differing fitnesses.

### Experimental designability phase diagrams for influenza antigens.

The phase diagrams from theory qualitatively match the results from our original numerical FLD paper [14], where we used force field-based binding energies and support vector machines to construct simulated phase diagrams. We now show that the analytical theory also agrees with *in vitro* experimental results.

Ref. [27] reports experimental *in vitro* binding affinity measurements between $M=2^{16}=65,536$ mutational variants of the human CH65 antibody and each of three mutational variants of the influenza H1 glycoprotein antigen. They chose L=16 amino acid sites which varied between the unmutated common ancestral antibody and the affinity-matured CH65 antibody and performed all combinatorial mutations between those two antibody types, meaning two possible amino acids at each of the 16 sites were tested. Their binding affinity measurements, obtained using the Tite-Seq teqchnique [28], are reported as log-dissociation constants $K_{d}(s)\equiv e^{\beta \Delta G_{\text{Ab}}(s)}$, where s is the antigen sequence label referring to one of three influenza strains: A/Massachusetts/1/1990 (MA90), A/Solomon Islands/3/2006 (SI06), and A/Massachusetts/1/1990 with the G189E mutation (G189E). Non-binding sequences were assigned the titration boundary value $-\log K_{d}=6$. After filtering out poor quality measurements, their published dataset contained collectively over 193, 000 binding affinities across the three antigens and all antibodies.

Here, we use ref. [27]’s rich dataset of reported binding affinities to construct the first-ever experimental FLD phase diagrams for each pair of influenza antigens, allowing direct comparison to our analytical calculations. Since host-antigen binding affinity measurements are unavailable, we work within the approximations that the three strains roughly equally bind the host receptor (ρ≈1) and that host concentrations are large $\left([\text{H}]\gg C_{0}\right)$. This approximation yields $F_{1}^{\text{free}}=F_{2}^{\text{free}}\approx 1$, rescaling the rectangular region bounded by the dotted lines in Figure 2 to the unit square, which effectively renormalizes the fitnesses of the two antigens to the range (0, 1]. For each tested antibody, and for each pair of influenza antigens, we use the experimental binding affinity measurements from ref. [27] to calculate Δ and substitute it into eq. (5), which is plotted on the unit square. Theoretical phase diagram boundaries are calculated by computing the means, standard deviations, and Pearson correlations of the two-antigen $\left(\Delta G_{\text{Ab}}\left(s_{1}\right),\Delta G_{\text{Ab}}\left(s_{2}\right)\right)$ joint distributions and substituting into eq. (9).

**FIG. 3.**
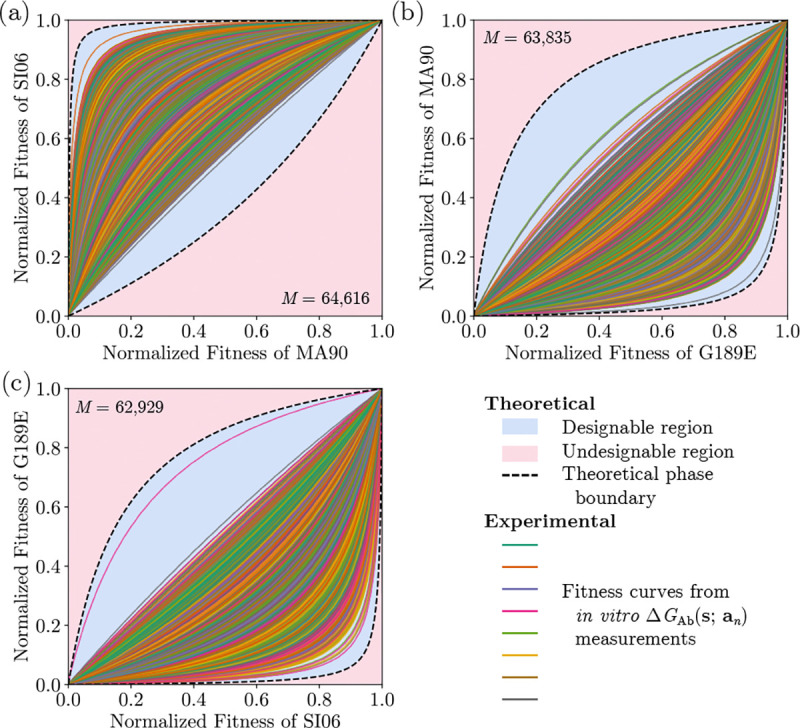
Experimental FLD phase diagrams from binding affinity measurements of over 62,000 antibodies with three influenza glycoprotein antigens, and theoretical phase boundaries (black, dashed) from the Gaussian approximation eq. (9). Empirical measurements are shown as solid, colorful lines.

These experimental results and theoretical boundaries are plotted in Figure 3. The solid lines of various colors are the transformed experimental data, while the black dashed lines indicate the theoretical boundaries separating the predicted designable (blue) and undesignable (red) regions. Figure 3(a) shows that the fitness of SI06 is generally higher than the fitness of MA90, with the designable region spanning most of the upper triangle above the central diagonal of the unit square. Figure 3(b) and (c) have their designable regions primarily spanning the lower triangle of the unit square, with SI06 generally having equal or higher fitness than G189E, which tends to have equal or higher fitness than MA90. Notably, each of the three pairs of antigens showcase one or more prominent “outlier” antibodies: orange top line in Figure 3(a), green and brown top lines as well as gray bottom line in Figure 3(b), and the pink top line in Figure 3(b). These antibodies push the designability frontier by being more selective for the antigen which generally binds more weakly to the other antibody variants. A notable feature of all three figure panels is the tightness of the theoretical bounds from eq. (9) near these outlier antibodies.

The lower bound in Figure 3(a) and the upper bound in Figure 3(b) appear overly optimistic. This is likely due to the non-Gaussian distribution of the log dissociation constants in ref. [27]’s dataset. One contributing factor to non-Gaussianity is the assignment of $-\log K_{d}=6$ to all antibody-antigen pairs which bind too weakly to be measured using Tite-Seq [27]. Another likely factor is the effect of evolutionary selection on the available antigens and antibodies. MA90 circulated in 1990 while SI06 circulated in 2006; its fitness may be expected to be higher than MA90 for most antibodies in the repertoire because it may indeed have evolved to escape related antibodies [27]. Similarly, G189E was specifically evolved in the laboratory to escape the universal common ancestral antibody, so its affinity to most of the antibodies in the repertoire are expected to be lower [27]. The extreme value statistics/REM results used in our theory rely on the $\Delta G_{\text{Ab}}(s)$ samples being unbiased, i.i.d. draws from the Gaussian distribution. Since both evolutionary selection and experimental choice of amino acids played a role in the construction of the antibody library, ref. [27]’s dataset is not expected to be Gaussian.

**FIG. 4.**
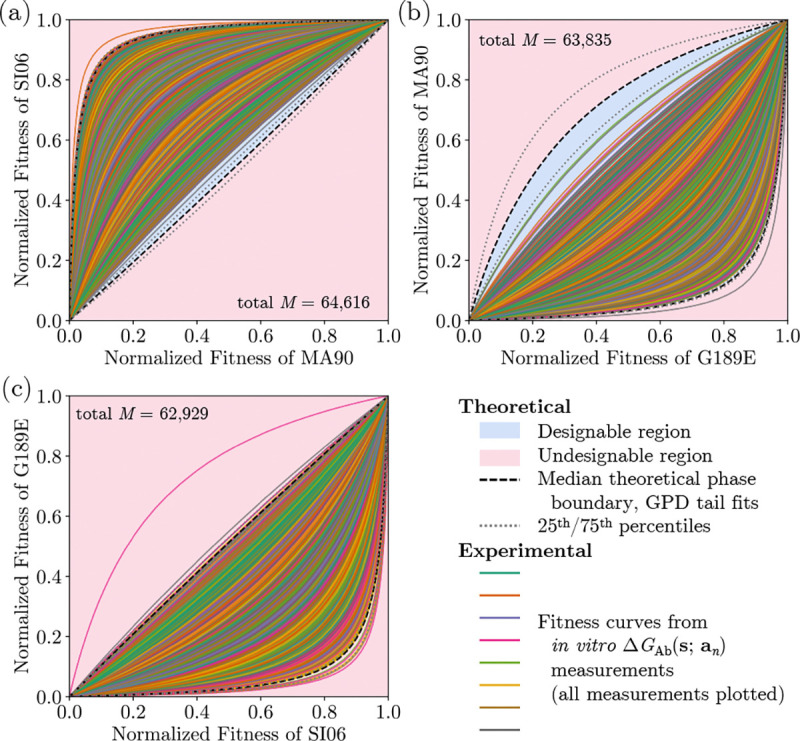
Experimental FLD phase diagrams from binding affinity measurements of over 62,000 antibodies with three influenza glycoprotein antigens, and semi-empiricial phase boundaries from fitting the tails of downsampled antibody datasets which are approximately 10% of the size of the original dataset. Empirical measurements are shown as solid, colorful lines. Estimated median phase boundaries from 10,000 independent downsampling trials are shown as black, dashed lines, and 25th/75th percentile phase boundaries are shown as gray, dotted lines.

In the End Matter, we address non-Gaussianity of the free energy difference $\Delta =\Delta G_{\text{Ab}}\left(s_{1}\right)-\Delta G_{\text{Ab}}\left(s_{2}\right)$ distribution by capturing tail behaviors with generalized Pareto distributions, since only tails behaviors impact extreme value results with finite samples. We subsequently show that this tail-fitting approach enables *scalability* of phase boundary prediction from a smaller set of experimental affinity measurements. End Matter Figure 4 shows that high-fidelity phase diagrams estimated for antibody libraries of size M can be obtained from only a training set of size 10%×M.

### Discussion.

In summary, we have introduced analytical foundations for biophysical FLD to customize the evolutionary fitnesses of different protein sequences, grounding our numerical work [14] in a theoretical basis. By working in the two-sequence, one-antibody case, we have found analytically tractable closed-form expressions for the boundaries of the designable region of the designability phase diagrams. We quantitatively justified the intuition that the area of the designable region—the codesignability score—should increase when the antibody-antigen binding free energy distributions for different antigens are decorrelated. Lastly, we validated the theoretical bounds on the designability phase diagrams by employing experimental *in vitro* binding affinities for over 62, 000 antibodies across three influenza antigens. Our theoretical bounds demonstrated successful ability to capture outlier antibodies which are responsible for expanding the designability frontier. We also showed that the theoretical bounds could be improved upon by fitting the tails of the free energy difference distributions directly, even when the antibody dataset was downsampled by 90%.

FLD is now building a solid experimental foundation, though there are current limitations and many directions for future efforts. In recent years, biophysical fitness formulas of the form in eq. (1) used as the basis of FLD have been validated *in vitro* [20] and with epidemiological data [21]. Now, the present work uses experimental binding affinity measurements [27] to support the notion that the space of all possible fitness landscapes separates into designable and undesignable regions, enabling the design of custom fitness landscapes for protein evolution—an idea we first proposed in ref. [14]. Although experimental binding affinities are substituted into an experimentally validated fitness formula, a limitation of this study is that true end-to-end experimental validation of FLD should involve performing direct fitness measurements of different strains in bulk culture in the presence of FLD-designed antibodies. This is a focus of our ongoing work.

Biophysical FLD opens the door not only to improved pandemic preparedness and biosecurity via proactive vaccine design [14] by discovering proactive antibodies that simultaneously inhibit wildtype and escape antigens, but also to possible cancer therapeutics such as engineered CAR-T cells or small peptides which may prevent immune escape of cancer cells. The widespread use of phages for directed evolution [29–31] is also an active target for applications of FLD; we suggest that FLD principles may be used for building experimental toolkits to engineer custom fitness landscape environments for experimental evolution and fundamental population genetics research.

## Supplementary Material

Supplement 1

## END MATTER

### Proof that codesignability score monotonically decreases with Pearson correlation $r_{12}$

Starting from eq. (10), we note

(13)
$$\begin{array}{l} \frac{\partial \text{CDS}}{\partial \sigma_{\Delta }}=\int_{0}^{F_{1}^{\text{free}}}\text{d}F_{1}\left[\frac{\partial F_{2}^{+}\left(F_{1}\right)}{\partial \sigma_{\Delta }}-\frac{\partial F_{2}^{-}\left(F_{1}\right)}{\partial \sigma_{\Delta }}\right].\\ =\int_{0}^{F_{1}^{\text{free}}}\;\text{d}F_{1}\left(\frac{\beta y_{M}}{F_{1}}-\frac{\beta y_{M}}{F_{1}^{\text{free}}}\right)\left[\frac{e^{\beta \Delta_{-}}}{F_{2}^{+}{\left(F_{1}\right)}^{2}}+\frac{e^{\beta \Delta_{+}}}{F_{2}^{-}{\left(F_{1}\right)}^{2}}\right]. \end{array}$$

Since $0<F_{1}\leq F_{1}^{\text{free}}$, the term within the parentheses is positive. Since exponentials in the numerators and quadratic terms in the denominators are also positive, the entire integrand is positive, which means $\partial \text{CDS}/\partial \sigma_{\Delta }>0$. Noting that $\partial \sigma_{\Delta }/\partial r_{12}=-\sigma_{1}\sigma_{2}/\sigma_{\Delta }<0$, it follows from chain rule that

(14)
$$\frac{\partial \text{CDS}}{\partial r_{12}}=\frac{\partial \text{CDS}}{\partial \sigma_{\Delta }}\frac{\partial \sigma_{\Delta }}{\partial r_{12}}<0,$$

which completes the proof.

### Experimental scalability of phase diagram prediction with tail-fitting and extreme value theory

To address non-Gaussianity, we also construct semiempirical phase diagrams by fitting the tails of the distribution for the free energy difference, $\Delta =\Delta G_{\text{Ab}}\left(s_{1}\right)-\Delta G_{\text{Ab}}\left(s_{2}\right)$, for each pair of antibodies. In extreme value theory, only the tails of the distribution and the sample size (i.e. antibody library size M) affect the expected extrema. The theoretical phase diagrams from the Gaussian distribution are constructed by fitting only two parameters: the mean $\mu_{\Delta }$ and standard deviation $\sigma_{\Delta }$. In Supplementary Appendix A, we show that by fitting each upper and lower tail using the generalized Pareto distribution (GPD) with three parameters per tail—still small compared to the number of samples—we can capture the empirically observed phase boundaries nearly perfectly (Supplementary Appendix A; Figure S4). This is expected, since the GPD fits are influenced by extremal values in the data distribution.

However, we find remarkably that even after massively downsampling the antibody dataset by ∼ 90%, fitting the tails to the subsampled distribution are still predictive of the phase boundaries for the full antibody dataset containing over 62,000 antibodies, as shown in Figure4 (see Supplementary Appendix A for step-by-step construction). We downsampled the complete antibody dataset to 10% of its original size in 10,000 independent trials and computed the phase boundaries. Figure 4 shows25th, 50th(median), and75thpercentilesforthesephase boundaries aggregated over the 10,000 trials. All predicted median phase boundaries (Figure 4: black, dashed lines) are tighter than the ones from the Gaussian approximation in Figure 3, despite having been constructed from roughly 10% of the original data. However, any extreme value prediction which relies on a smaller set of finite samples is less likely to capture outliers which may strongly impact the GPD fits, as seen in Figure 4(c), where the pink antibody outlier is not captured by the phase boundaries from the downsampled fits. The quartile phase boundaries (Figure 4: gray, dotted lines) show that some boundary predictions are better than others, depending on the presence/absence of outliers drawn from the antibody library (see Figure S7 for complete distributions of expected extrema from the 10,000 trials). Using larger antibody libraries for tail fitting fixes these discrepancies, as mentioned previously (Supplementary Appendix A; Figure S4). Overall, the downsampling approach shows promise for laboratory scalability of FLD phase diagram construction by reducing the number of antibodies needed to calculate boundaries, provided that tails are captured aptly.

We also emphasize that the phase diagrams computed here and in the main text are limited in that completely random antibody sequence sampling was not conducted, and only around $2^{16}\;\sim \;6.6\times 10^{4}$ out of $20^{16}\;\sim \;6.6\times 10^{20}$ possible antibody mutations at the L=16 sites were explored, so broader amino acid variation as well as expansion of the paratope sites may yield expanded designable (blue) regions. Also, we add that eq. (1) suggests that apparent “gaps” between the outlier antibodies and the bulk of the distribution (e.g. between the pink outlier and bulk in Figure 4(c)) may be explored by using combinations of antibodies at different concentrations—an idea supported by our random antibody ensemble sampling results in the original FLD paper [14].
