# Supplementary material for: Phase diagrams for biophysical fitness landscape design
Source: bioRxiv. 2026 Mar 20:2025.09.10.675274. Preprint. [Version 2] doi: 10.1101/2025.09.10.675274 (PMC13015304; doi:10.1101/2025.09.10.675274)
Supplement: Supplement 1 [file NIHPP2025.09.10.675274v2-supplement-1.pdf]

bioRxiv preprint doi: https://doi.org/10.1101/2025.09.10.675274; this version posted March 20, 2026. The copyright holder for this preprint (which was not certified by peer review) is the author/funder, who has granted bioRxiv a license to display the preprint in perpetuity. It is made available under aCC-BY 4.0 International license.

Supplementary Information for "Phase diagrams for biophysical fitness landscape design"

Vaibhav Mohanty[1,2,3,*] and Eugene I. Shakhnovich[1,*]
[1] *Department of Chemistry and Chemical Biology, Harvard University, Cambridge, MA 02138*
[2] *Harvard/MIT MD-PhD Program, Harvard Medical School, Boston, MA 02115 and Massachusetts Institute of Technology, Cambridge, MA 02139*
[3] *Program in Health Sciences and Technology, Harvard Medical School, Boston, MA 02115 and Massachusetts Institute of Technology, Cambridge, MA 02139*
[*] Correspondence: mohanty@hms.harvard.edu (V.M.); shakhnovich@chemistry.harvard.edu (E.I.S.).

APPENDIX A: CONSTRUCTING SEMI-EMPIRICAL PHASE DIAGRAMS FROM TAIL FITTING

Here, we construct semi-empirical FLD phase diagrams for the three pairs of influenza antigens without using the Gaussian approximation. Instead, we fit the tails of the distribution of free energy differences in order to obtain more accurate boundary values from extreme value statistics. Finally, we show that downsampling the antibody dataset by 90% still yields tail fits which are predictive of the phase boundaries for the full antibody dataset.

Binding affinity distributions

First, in Figure S1 we reproduce the $\log K_d$ distributions (equivalent to the ΔG distributions) for the three influenza antigens, where each datapoint represents one of up to up to $65,535$ antibodies whose binding affinities to each antigen were measured by [27]. The distributions are directly reproduced from the dataset; not all antibodies have binding affinites reported (and are stored as NaN values), which is why each antigen has a different number of antibody datapoints. Notably, the Tite-seq technique [28] used by [27] was limited to $\log K_d$ values stronger (i.e. more negative) than -6, so antibodies which bound more weakly (i.e. $\log K_d \geq -6$) were automatically assigned a value of -6, resulting in large spikes in two of the antigens' binding affinity distributions (Figure S1(a-b)).

Binding affinity difference distributions

Next, we compute the differences between each antibody's $\log K_d$ for pairs of antigens:

$$\log K_{d,1} - \log K_{d,2} = \beta[\Delta G(\mathbf{s}_1) - \Delta G(\mathbf{s}_2)]. \tag{15}$$

These difference distributions are plotted in Figure S2. As can be readily seen in the histograms, these distributions are non-Gaussian, so the approximation applied in the main text is not expected to hold necessarily. To obtain better estimates of phase diagram boundaries (relative to those obtained from the Gaussian approximation), we only need to capture the tail behaviors for each distribution.

Extreme value theory for expected bound on distribution tails with finite samples

In extreme value statistics, the generalized Pareto distribution (GPD) is commonly used for this tail fitting task [32, 33], and it enables an analytically tractable estimate for the expectation of the tail's maximum for a certain number of samples. The GPD's probability density function is given by

$$p(x;\xi,\mu,\sigma) = \frac{1}{\sigma}\left(1 + \frac{\xi(x-\mu)}{\sigma}\right)^{-(1+1/\xi)}, \tag{16}$$

where μ is called the location parameter (*not* necessarily equal to the distribution's mean), σ is the scale parameter, and ξ is the shape parameter. Following standard extreme value theory [32, 33], we first construct a cumulative distribution function for a random variable $X = \log K_{d,1} - \log K_{d,2}$ which represents a datapoint drawn from the tail of the binding affinity difference distribution modeled by the GPD. The location parameter is set to $\mu = 0$ because the distribution will be shifted through the

bioRxiv preprint doi: https://doi.org/10.1101/2025.09.10.675274; this version posted March 20, 2026. The copyright holder for this preprint (which was not certified by peer review) is the author/funder, who has granted bioRxiv a license to display the preprint in perpetuity. It is made available under aCC-BY 4.0 International license.

introduction of a threshold. As is commonly done in extreme value theory, we shift the data distribution so that the tail begins at a threshold value u, which is moved to the origin. So, we define $Y = X - u$, and the CDF can be written

$$\mathbb{P}(Y \leq y \,|\, Y > 0) = \int_0^y \mathrm{d}y' \, \frac{1}{\sigma}\left(1 + \frac{\xi y'}{\sigma}\right)^{-(1+1/\xi)} = 1 - \left(1 + \frac{\xi y}{\sigma}\right)^{-1/\xi}. \tag{17}$$

Undoing the affine shift, we can write

$$\mathbb{P}(X - u \leq x \,|\, X > u) = 1 - \left(1 + \frac{\xi(x-u)}{\sigma}\right)^{-1/\xi}. \tag{18}$$

The probability of a datapoint being greater than a certain value x *within the tail* is given by the product of probabilities of drawing a datapoint from the tail the conditional probability above:

$$\mathbb{P}(X > x) = \mathbb{P}(X - u > x \,|\, X > u)\mathbb{P}(X > u) = q\left(1 + \frac{\xi(x-u)}{\sigma}\right)^{-1/\xi}, \tag{19}$$

where we let $q \equiv \mathbb{P}(X > u)$ be a pre-defined threshold quantile to which fixes the start of the tail.

Now, we define

$$X_{\max} \equiv \max\{X_1, \ldots, X_M\} \tag{20}$$

to be a random variable representing the maximum of M i.i.d. datapoints. The maximum will be less than some value x only if all drawn variables are also less than x:

$$\begin{aligned}\mathbb{P}(X_{\max} \leq x) &= \prod_{i=1}^{M} \mathbb{P}(X_i \leq x) = [\mathbb{P}(X \leq x)]^M \\ &= [1 - \mathbb{P}(X_{\max} > x)]^M \\ &= \left[1 - q\left(1 + \frac{\xi(x-u)}{\sigma}\right)^{-1/\xi}\right]^M.\end{aligned} \tag{21}$$

In the large M (many samples) limit, we take the typical Poisson limit commonly invoked for independent events:

$$\mathbb{P}(X_{\max} \leq x) = \left[1 - q\left(1 + \frac{\xi(x-u)}{\sigma}\right)^{-1/\xi}\right]^M \approx \exp\left[-Mq\left(1 + \frac{\xi(x-u)}{\sigma}\right)^{-1/\xi}\right]. \tag{22}$$

The CDF above defines a generalized extreme value (GEV) distribution from which we can quickly compute the expectation of the extremal value by integrating within the tail:

$$\mathbb{E}[X_{\max}] = \int_u^\infty \mathrm{d}x \, x \mathbb{P}(X_{\max} = x). \tag{23}$$

The PDF is computed by taking a derivative of the CDF with respect to x:

$$\mathbb{P}(X_{\max} = x) = \frac{1}{\sigma} Mq \left(1 + \frac{\xi(x-u)}{\sigma}\right)^{-1-\frac{1}{\xi}} \exp\left[-Mq\left(1 + \frac{\xi(x-u)}{\sigma}\right)^{-1/\xi}\right]. \tag{24}$$

Performing the substitution

$$t = Mq\left(1 + \frac{\xi(x-u)}{\sigma}\right)^{-1/\xi} \tag{25}$$

also gives us

$$x = u + \frac{\sigma}{\xi}\left((Mq)^{\xi} t^{-\xi} - 1\right), \tag{26}$$

bioRxiv preprint doi: https://doi.org/10.1101/2025.09.10.675274; this version posted March 20, 2026. The copyright holder for this preprint (which was not certified by peer review) is the author/funder, who has granted bioRxiv a license to display the preprint in perpetuity. It is made available under aCC-BY 4.0 International license.

and

$$\mathrm{d}x = -\sigma(Mq)^{\xi} t^{-\xi-1}\,\mathrm{d}t\,. \tag{27}$$

Now, the integral becomes

$$\begin{aligned}\mathbb{E}[X_{\max}] &= \int_{t=Mq}^{0} \mathrm{d}t\,\frac{-\sigma(Mq)^{\xi}t^{-\xi-1}}{\sigma(Mq)^{\xi}} t^{\xi+1}\left[u+\frac{\sigma}{\xi}\left((Mq)^{\xi}t^{-\xi}-1\right)\right]e^{-t}\\ &= \int_{0}^{Mq}\mathrm{d}t\left[u+\frac{\sigma}{\xi}\left((Mq)^{\xi}t^{-\xi}-1\right)\right]e^{-t}.\end{aligned} \tag{28}$$

In the limit of large M (which we used in the Poisson limit earlier), we take $Mq \to \infty$, so

$$\begin{aligned}\mathbb{E}[X_{\max}] &\approx \int_0^{\infty}\mathrm{d}t\,ue^{-t} + \frac{\sigma}{\xi}\int_0^{\infty}\mathrm{d}t\left((Mq)^{\xi}t^{-\xi}-1\right)e^{-t}\\ &= u+\frac{\sigma}{\xi}\left[-1+(Mq)^{\xi}\int_0^{\infty}\mathrm{d}t\,t^{-\xi}e^{-t}\right]\\ &= u+\frac{\sigma}{\xi}\left[-1+(Mq)^{\xi}\Gamma(1-\xi)\right],\end{aligned} \tag{29}$$

where we have used the Gamma function Γ and have assumed $\xi < 1$, which we will find later holds for all of the datasets studied here. By fitting the GPD PDF (eq. (16)) with $\mu = 0$ to the tails of our log K_d difference distributions, we can obtain estimates for σ and ξ and then substitute them into eq. (29) in order to predict the expected bound on the data distribution for finite samples M. For an antibody library of size M, this is perhaps tautological, but below we will also show that we can downsample the antibody library to size $0.10 \times M$ and still make predictions on the boundaries for the full library of size M.

Fitting tails of binding affinity difference distributions

We now return to our empirical study of the binding affinity distributions. Although various methods exist for deciding where tail thresholds should be optimally placed [32, 33], we choose threshold quantiles arbitrarily to be the extremal 5% of the data distribution on either tail, *except* for the lower tail of the $\log K_{d,\mathrm{SI06}} - \log K_{d,G189E}$ distribution, for which we choose the bottom 20% in order to capture the large spike which is caused by the Tite-seq detection boundary.

After truncation based on the quantile thresholds described above, we reflect and/or affine shift the tails so that they are all in the non-negative real domain. Then, we fit the GPD to the tails using maximum likelihood estimation, as pre-implemented using SciPy's `scipy.stats.genpareto.fit` [34]. From the estimated $\hat{\xi}$ and $\hat{\sigma}$ values, we calculated the expected bounds on the tails using eq. (29). In Figure S3, we show the tails with the GPD fits, the estimated bounds from extreme value theory, and the actual experimentally observed bounds; the tails have been re-reflected and re-shifted to their original domains and orientations.

Semi-empirical phase diagrams without the Gaussian approximation: tail fitting

Substituting the estimated bounds from eq. (29) into main text eq. (9), we are able to construct semi-empirical phase diagrams (Figure S4) as an alternative to the phase diagrams computed from the Gaussian approximation in main text Figure 3. Compared to the Gaussian approximation, capturing the tail behaviors accurately yield excellent theoretical estimates. This is to be expected since we are essentially using the extremal data values from the empirical distribution to estimate the boundary, albeit somewhat indirectly through fitting ξ and σ.

A more powerful consequence of this tail fitting-based approach, however, is that *experimental scalability* is enabled, which we subsequently investigate by downsampling the antibody library.

Scalability of phase boundary inference: downsampling the antibody library

We now downsample the antibody library to roughly 10% of its original size. We randomly choose 10% of the 65,535 indices; however, some antibodies have NaN reported as their binding affinity to particular antigens. So, the exact number of antibodies comprising the downsampled binding affinity difference distributions (Figure S5) is not exactly 10% of the full distributions from

bioRxiv preprint doi: https://doi.org/10.1101/2025.09.10.675274; this version posted March 20, 2026. The copyright holder for this preprint (which was not certified by peer review) is the author/funder, who has granted bioRxiv a license to display the preprint in perpetuity. It is made available under aCC-BY 4.0 International license.

Figure S2. In general, the sample size of 10% captures the general shape of the histogram of the full distribution, but of course outliers are less likely to be sampled.

We then isolate tails using the same quantile cutoffs used for the full distribution, reflect and/or affine shift, and fit the GPD. Then, we calculate the expected phase diagram boundary using eq. (29), but with M set to the *full* antibody dataset size (i.e. between 62,000 and 65,000, depending on how many NaN values were present). This means we are using the fitted parameters from the downsampled dataset but are *predicting* the phase boundaries for the full datset. Example tail fits from a single trial of the sampling procedure are shown in Figure S6. The downsampling procedure is conducted for 10,000 trials, and the distributions for the expected extrema $\mathbb{E}[\min(\log K_{d,1} - \log K_{d,2})]$ (for lower tails) or $\mathbb{E}[\max(\log K_{d,1} - \log K_{d,2})]$ (for upper tails), constructed from those 10,000 trials, are shown in Figure S7, along with quartile boundaries, means, and the true extrema for comparison. The phase diagrams arising from the downsampling procedure are shown and discussed in main text Figure 4, with quartile boundaries plotted as well. The ability to use a smaller antibody sample to predict the phase boundaries for the full antibody library demonstrates the scalability of the experimental approach for elucidating FLD phase diagrams.

However, it should be noted that the GPD fits and predicted maxima are heavily dependent on outlier values. As we see in the main text, sometimes the phase boundary is underestimated if outliers are not captured in a particular sample. This is naturally always a limitation of extreme value prediction using smaller samples.

Supplementary Figures

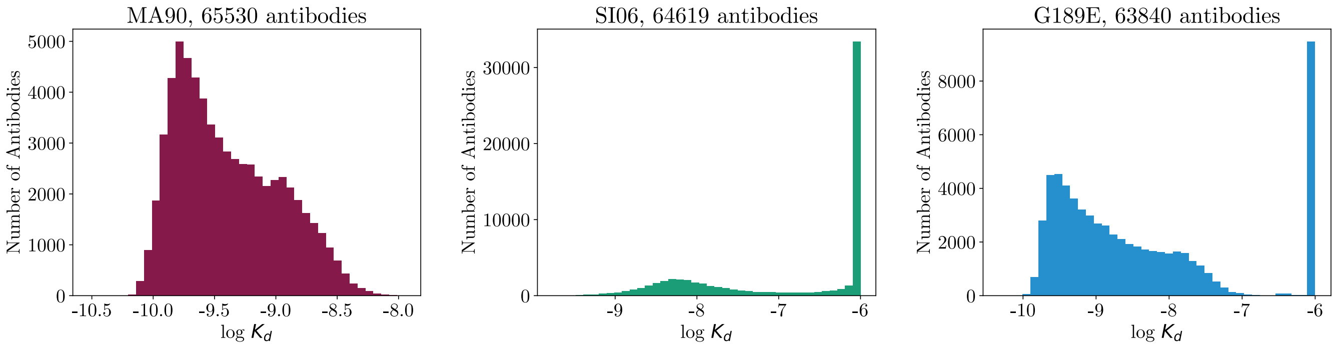

FIG. S1. Distribution of log dissociation constants between over 62,000 antibodies and each of three influenza antigens, directly reproduced from the public dataset from [27]. Spikes in the distribution are due to weak binders whose $\log K_d \geq -6$ being binned to a value of -6.

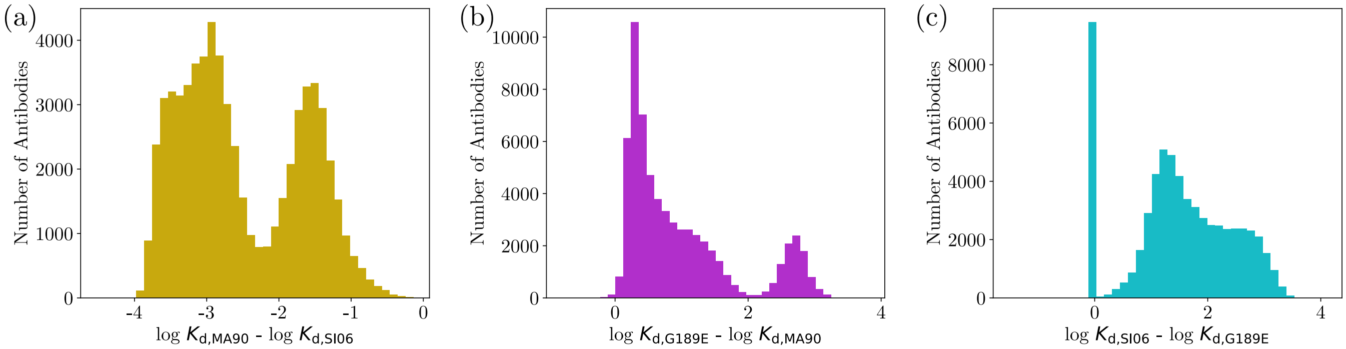

FIG. S2. Distributions of the difference in binding affinity each antibody has to two different influenza antigens, as described by eq. (15). Antibody count M differs for each distribution based on the number of NaN values in the dataset. (a) $M = 64,616$, (b) $M = 63,835$, and (c) $M = 62,929$.

bioRxiv preprint doi: https://doi.org/10.1101/2025.09.10.675274; this version posted March 20, 2026. The copyright holder for this preprint (which was not certified by peer review) is the author/funder, who has granted bioRxiv a license to display the preprint in perpetuity. It is made available under aCC-BY 4.0 International license.

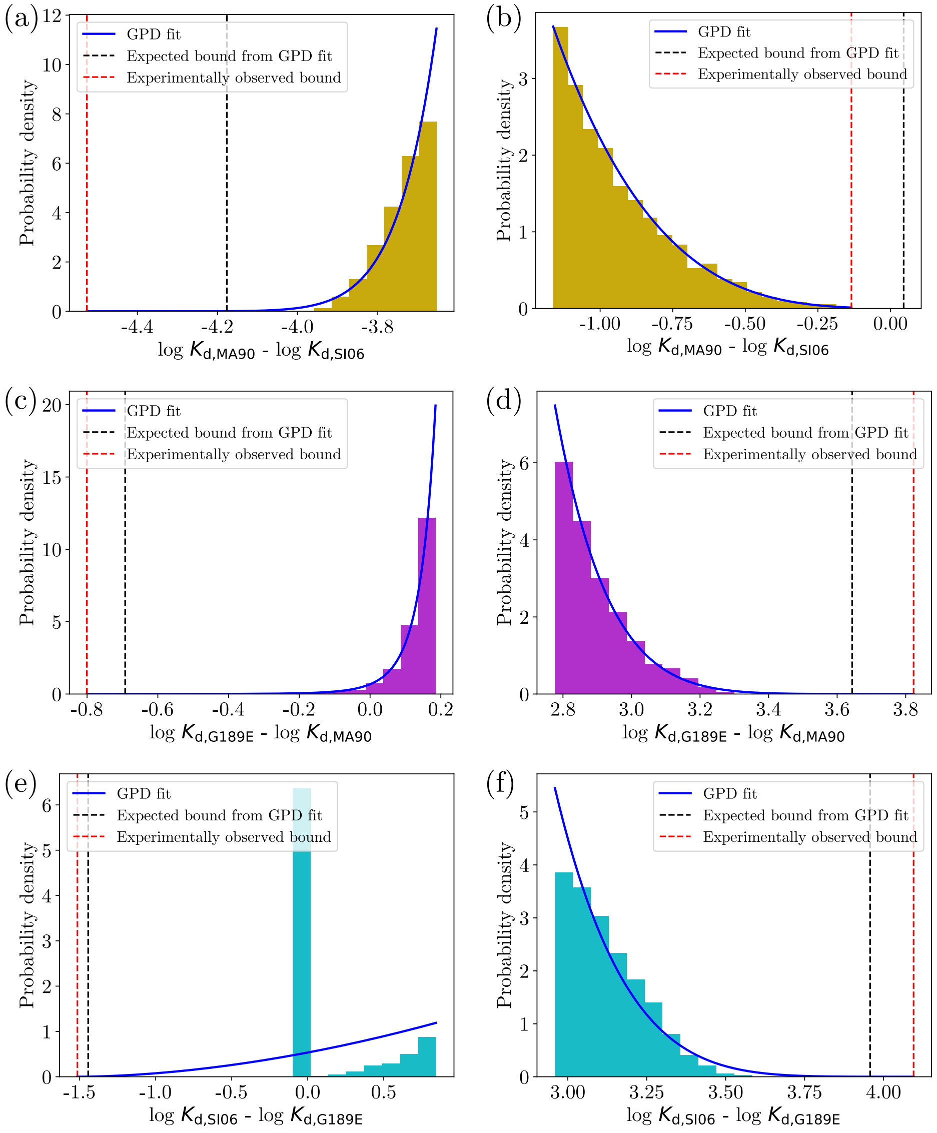

FIG. S3. Upper and lower tails of the distributions in Figure S2 fit using the generalized Pareto distrbution in eq. (16), with location parameter set to $\mu = 0$. (a) Lower tail of Figure S1(a), (b) upper tail of Figure S1(a), (c) lower tail of Figure S1(b), (d) upper tail of Figure S1(b), (e) lower tail of Figure S1(c), (f) upper tail of Figure S1(c). 5% quantiles were used to threshold the tails for (a-d,f), and the lower 20% quantile was used to threshold the tail for (e), in order to capture the spike.

bioRxiv preprint doi: https://doi.org/10.1101/2025.09.10.675274; this version posted March 20, 2026. The copyright holder for this preprint (which was not certified by peer review) is the author/funder, who has granted bioRxiv a license to display the preprint in perpetuity. It is made available under aCC-BY 4.0 International license.

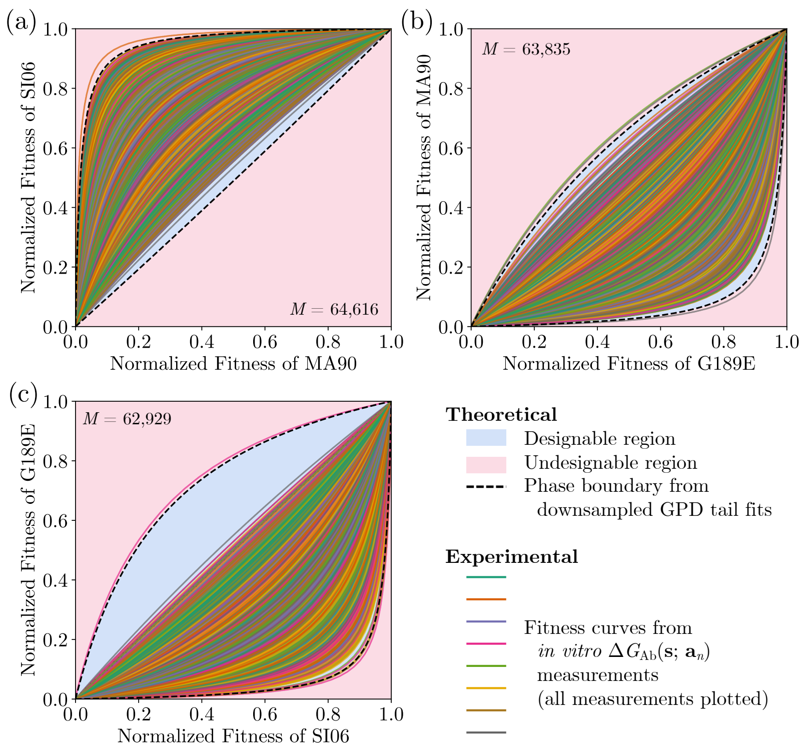

FIG. S4. Semi-empirical phase diagrams constructed by substituting the estimated bounds on the binding affinity difference distributions (i.e. black dashed lines in fig. S3) into main text eq. (9). The theoretical phase boundaries show excellent agreement with the experimental extremal antibodies, as expected since the full data range is used to estimate the bounds.

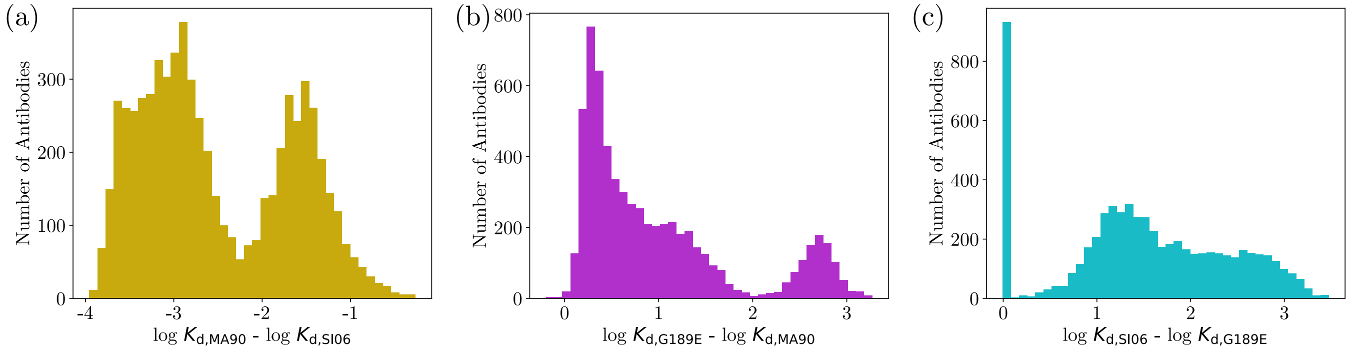

FIG. S5. A representative example of binding affinity difference distributions for a single downsampling trial, using approximately 10% of the original antibody library. Antibody count M differs for each distribution (and may not be exactly 10% of the original datasets) based on the number of NaN values in the dataset. (a) $M = 6,469$, (b) $M = 6,396$, and (c) $M = 6,314$.

bioRxiv preprint doi: https://doi.org/10.1101/2025.09.10.675274; this version posted March 20, 2026. The copyright holder for this preprint (which was not certified by peer review) is the author/funder, who has granted bioRxiv a license to display the preprint in perpetuity. It is made available under aCC-BY 4.0 International license.

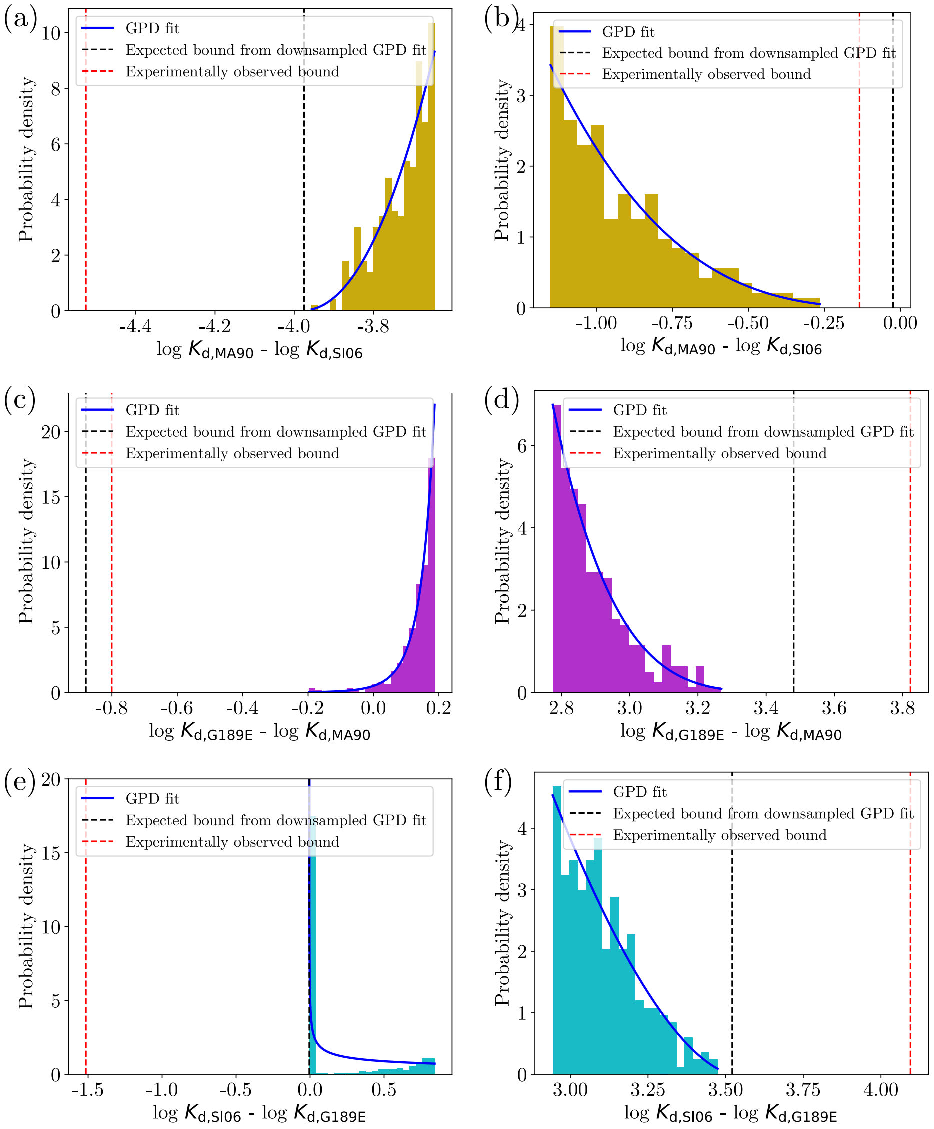

FIG. S6. Upper and lower tails of the distributions in Figure S5 fit using the generalized Pareto distrbution in eq. (16), with location parameter set to $\mu = 0$. (a) Lower tail of Figure S5(a), (b) upper tail of Figure S5(a), (c) lower tail of Figure S5(b), (d) upper tail of Figure S5(b), (e) lower tail of Figure S5(c), (f) upper tail of Figure S5(c). 5% quantiles were used to threshold the tails for (a-d,f), and the lower 20% quantile was used to threshold the tail for (e), in order to capture the spike.

bioRxiv preprint doi: https://doi.org/10.1101/2025.09.10.675274; this version posted March 20, 2026. The copyright holder for this preprint (which was not certified by peer review) is the author/funder, who has granted bioRxiv a license to display the preprint in perpetuity. It is made available under aCC-BY 4.0 International license.

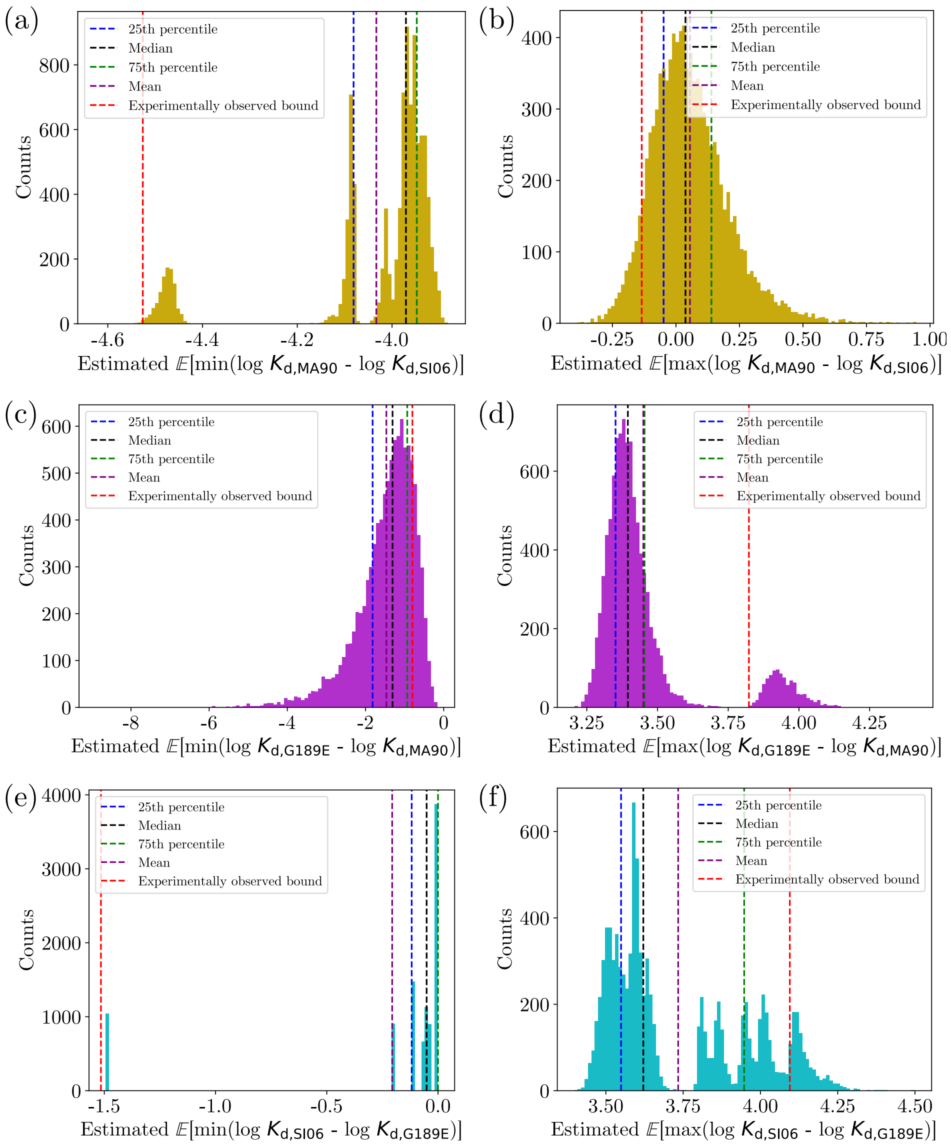

FIG. S7. Distributions for the estimated extremal values for each tail, where each distribution has been built from 10,000 trials of the downsampling procedure. For lower tails, $\mathbb{E}[\min(\log K_{d,1} - \log K_{d,2})]$ is calculated for antigens 1 and 2, and for upper tails $\mathbb{E}[\max(\log K_{d,1} - \log K_{d,2})]$ is calculated for antigens 1 and 2. (a) Lower tail for MA90 vs. SI06, (b) upper tail for MA90 vs. SI06, (c) lower tail for G189E vs. MA90, (d) lower tail for G189E vs. MA90, (e) lower tail for SI06 vs. G189E (f) upper tail for SI06 vs. G189E. Vertical lines indicate 25th, 50th (i.e. median), and 75th percentiles as well as the empirical mean and the experimentally observed extrema.
